# CRISPR/Cas9-based discovery of ccRCC therapeutic opportunities through molecular mechanism and immune microenvironment analysis

**DOI:** 10.3389/fimmu.2025.1619361

**Published:** 2025-07-10

**Authors:** Bo Han, Weiyang Liu, Wanhui Wang, Zhuolun Li, Bosen You, Dongze Liu, Yunfeng Nan, Tiankai Ding, Zhou Dai, Yantong Zhang, Wei Zhang, Qing Liu, Xuedong Li

**Affiliations:** Department of Urology, The Second Affiliated Hospital of Harbin Medical University, Harbin, China

**Keywords:** CRISPR-Cas9 screening, ccRCC, prognostic model, MELK, immunotherapy

## Abstract

**Introduction:**

Clear cell renal cell carcinoma is a common and aggressive form of renal cell carcinoma. Its incidence continues to rise, and metastatic recurrence leads to poor clinical outcomes. Current prognostic biomarkers lack reliability. We integrated multi-omics data to discover key ccRCC genes and build a prognostic model to improve risk prediction and guide treatment decisions.

**Methods:**

Our study integrated genome-wide CRISPR screening data from DepMap and transcriptomic profiles from TCGA to identify key genes associated with ccRCC pathogenesis. Initial screening identified 11 candidate genes through differential expression analysis and CRISPR functional validation. Using LASSO and Cox regression, we selected five key genes (GGT6, HAO2, SLPI, MELK, and EIF4A1) for model construction. The functional role of MELK was tested by knockdown experiments. Additional analyses included tumor mutation burden, immune microenvironment assessment, and drug response prediction.

**Results:**

The model stratified patients into high-risk and low-risk groups with distinct survival outcomes. High-risk cases showed higher mutation loads, immunosuppressive features, and activated cytokine pathways, whereas low-risk cases displayed metabolic pathway activity. MELK knockdown reduced cancer cell proliferation and migration. High-risk patients exhibited better responses to targeted drugs such as pazopanib and sunitinib.

**Discussion:**

Our study demonstrates the pivotal role of MELK in ccRCC progression. This multi-omics-driven model elucidates MELK-mediated mechanisms and their interactions with the tumor microenvironment, providing novel strategies for risk stratification and targeted therapy. Future studies will validate these findings in independent cohorts and investigate the regulatory networks of MELK to identify potential therapeutic targets.

## Introduction

Renal cell carcinoma (RCC) ranks among the most prevalent cancers in the urological system, with its incidence on the rise, representing approximately 2%–3% of malignant neoplasms in adults (1). RCC is a prevalent malignancy within the genitourinary tract, characterized by its aggressive nature and high fatality rate (2). Among RCC subgroups, clear cell Renal Cell Carcinoma (ccRCC) predominates histologically, representing about 75-80% among RCC diagnoses (3). Globally, approximately 400,000 RCC diagnoses are identified each year, with the United States contributing an estimated 82,000 cases in 2024 with ccRCC accounting for about 75%–80% of these cases. RCC is responsible for over 170,000 deaths annually. The vast majority of which were ccRCC, with around 15,000 deaths attributed to the disease.

ccRCC exhibits significant heterogeneity, a high propensity for metastasis, and a generally unfavorable prognosis (4). Despite surgical excision being the mainstay treatment for patients with localized ccRCC, a significant proportion 30–40% of these patients experience metastatic relapse after surgery during subsequent follow-up. As a result, early detection of metastatic propensity in ccRCC is crucial for enhancing the precision of prognostic predictions. At present, our knowledge of the pathogenesis of ccRCC remains incomplete, and reliable tumor biomarkers for predicting prognosis have yet to be established.

Recently, high-throughput screening initiatives, such as the DepMap project, have gained prominence. These projects leverage RNA interference silencing and CRISPR-Cas9 (Clustered Regularly Interspaced Short Palindromic Repeats-associated protein 9) knockout techniques to pinpoint possible essential genes vital to tumor survival, metastasis, or recurrence (5–7). Researchers have employed CRISPR technology to selectively knock out target genes, thereby exploring potential therapeutic strategies (8, 9). To systematically identify potential cancer biomarkers, the CRISPR-Cas9 system has been employed to screen essential genes regulating cancer cell growth and viability. To enhance the specificity of CRISPR-based screens, the CERES algorithm was developed to computationally correct copy number effects, thereby quantifying the median impact of core and dispensable genes on a for each individual cell line basis (10). Genes deemed essential in a limited number of cell lines are regarded as more promising therapeutic targets, since targeting these genes is less likely to induce off-tissue toxicity. In addition, studying the prognostic value of ccRCC can help urologists better treat patients.

By combining DepMap CRISPR screening and TCGA transcriptomic data, we identified five pivotal ccRCC-associated genes. Using LASSO and multivariate Cox regression, we developed a prognostic model and analyzed its relationships with tumor mutational burden (TMB), Tumor microenvironment (TME) immune infiltration, immunotherapy response, and chemotherapy efficacy. A clinical nomogram incorporating risk scores and clinical features was established for ccRCC prognosis prediction.

## Method

### Data collection and preprocessing

This study focuses on characterizing molecular biomarkers while investigating potential therapeutic targets for ccRCC. Utilizing TCGA database, gene expression profiles and clinical data from 537 ccRCC patients were analyzed. Differential expression analysis was conducted between matched tumor-normal tissue pairs from the TCGA cohort, with differentially expressed genes (DEGs) identified using a false discovery rate (FDR) threshold of less than 0.05 and a log2 fold change (log2FC) greater than 1 as the criteria for defining primary cancer-associated genes. Subsequently, the DepMap database contains gene dependency data from cancer cell lines, was employed in conjunction with CRISPR-Cas9 gene-editing technology to further validate the critical role of these genes in cancer cell survival. For this purpose, the CRISPR dataset from the 24Q4 release of the DepMap database was downloaded, and genes with Chronos scores below zero were identified as essential genes. By integrating the analytical results from TCGA and DepMap, the study successfully identified a group of core genes closely associated with ccRCC, which may serve as potential diagnostic markers and pharmacological targets for further in-depth analysis. External validation was performed using the GEO dataset GSE26909 (n=39), with risk scores calculated using the same coefficients derived from the TCGA cohort.

### Identification of DEGs

After identifying 11 genes in ccRCC, we first analyzed their expression and copy number variation (CNV) profiles. A cutoff-based approach was applied, and heatmaps were generated using the “pheatmap” R package (11). Next, differential expression and co-expression analyses of these 11 genes were performed to assess their expression patterns. Boxplots were generated using the ‘ggpubr’ R package. (12).

### Recognition of key genes in ccRCC

To identify survival-related genes in ccRCC, we conducted univariate, LASSO-penalized, and multivariate Cox proportional hazards regression analyses using R’s glmnet package to develop a prognostic prediction model (13–16). The heatmap illustrates the pattern of clinical feature distribution across patients in the high-risk and low-risk groups which was generated to visualize the expression patterns of DEGs across the patient samples. The expression data were normalized and log2-transformed to reduce skewness and improve comparability. Hierarchical clustering was performed on both genes and samples to group those with similar expression profiles. The chord diagram was generated to visualize regulatory or functional interactions between the top DEGs. The risk score for each patient was calculated using a linear combination of the expression levels of the DEGs, weighted by their respective regression coefficients derived from multivariate Cox analysis. The formula is as follows:


Riskscore = ∑​iCoefficient (i)*Expression of gene(i)

Differences in survival between risk strata were evaluated through Kaplan-Meier (KM) analysis performed with the “survival” R package (17, 18). Patients were dichotomized into high- and low-risk groups using the median risk score as the threshold. This cutoff was selected to ensure balanced group sizes and clinical interpretability. Time-dependent Receiver operating characteristic (ROC) analysis evaluated the gene risk model’s performance using 1-year, 3-year, and 5-year follow-up data. We validated the optimal threshold value via principal component analysi (PCA) (19). Calibration curves approaching the 45-degree line indicated optimal predictive performance of the nomogram.

### Consensus clustering analysis

This study investigates the application of clustering analysis in data classification through experiments, centered on the k-means partitioning method and its implementation in the R environment using the ConsensusClusterPlus tool (20). The experiment employed Euclidean distance as the similarity measure and incorporated the Partitioning Around Medoids (PAM) algorithm to perform clustering analysis on the dataset, ranging from 2 to 9 clusters. the study constructed a reliable consensus matrix, significantly reducing inter-cluster overlap and achieving efficient data classification. This analysis was implemented using the R package ConsensusClusterPlus.

### Predictive nomogram with interactive dynamic features

We developed the prognostic nomogram with the “rms” package (21) and implemented an interactive web calculator using “shiny” and “DynNom” packages (22, 23) for real-time survival probability estimation. The model’s predictive performance was validated through calibration plots comparing observed KM versus predicted 1-year, 3-year, and 5-year survival outcomes.

### TMB calculation

TMB was quantified based on the count including nonsynonymous single nucleotide variants and insertion-deletion alterations per megabase. Leveraging the “maftools” R package, we derived TMB values for our predictive model (24).

### Function enrichment analysis

Gene Ontology (GO), Kyoto Encyclopedia of Genes and Genomes (KEGG), and Gene Set Enrichment Analysis (GSEA) were performed using the R packages clusterProfiler and GOplot to identify biological functions and pathways associated with cancer essentiality in high-risk vs low-risk groups (25). Results were visualized with ggplot2 (26).

### Drug sensitivity analysis

We conducted a drug sensitivity analysis aimed at evaluating the impact of various compounds on specific cell lines. For this purpose, we utilized the “limma”, “ggpubr” and the “pRRophetic” R package for our analysis, with the selection threshold set at p < 0.05 and q < 1 (27).

### Investigation of immune cell infiltration

Immune cell infiltration profiles were analyzed using complementary approaches: ssGSEA via the GSVA package quantified 22 immune cell subtypes, while CIBERSORT assessed immune infiltration patterns and their association with immune checkpoints across risk groups.

### Cell culture

The ccRCC cell lines 786O, 769P, and Caki-1 were obtained from the American Type Culture Collection (ATCC). The cells were cultured in RPMI-1640 medium containing 10% fetal bovine serum (FBS) and 1% penicillin/streptomycin. All reagents were purchased from Gibco (Invitrogen-Gibco). Cells were incubated at 37°C with 5% CO_2_ in a humidified environment.

### Human specimens

This study was conducted at the Second Affiliated Hospital of Harbin Medical University to provide a scientific basis for ccRCC early detection and therapy. Tumor and adjacent normal tissues (0.5 cm³ each) were collected from surgically treated ccRCC patients. The study was approved by the hospital’s Ethics Committee, after obtaining participant consent. Formalin-fixed paraffin-embedded specimens were prepared for immunohistochemistry, and clinical data were verified by two board-certified surgeons.

### Western blot

Cells were lysed in RIPA buffer containing protease inhibitors (Seven, China), collected by scraping (BIOFIL), and quantified by BCA (Beyotime). Proteins were separated by 10% SDS-PAGE, transferred to PVDF membranes (Millipore), and incubated with specific primary antibodies at 4°C for 12-16 hours followed by HRP-secondary antibodies (RT, 1 h) were detected by chemiluminescence (Tanon).

### Cell colony formation analyze

Cells were harvested in RIPA/protease inhibitor cocktail (Seven, China), collected by scraping (BIOFIL), and quantified by BCA (Beyotime). Proteins were resolved on 10% SDS-PAGE, transferred to PVDF membranes, and immunoblotted with primary antibodies (4°C, overnight) and HRP-secondaries (RT, 1 h), followed by chemiluminescent detection (Tanon). Following distilled water washes and air-drying, colonies (≥50 cells) were microscopically counted to calculate formation rates, with images captured for analysis.

### Transwell assay

Cells (5 × 10^4^ ccRCC) were seeded in serum-free 8 µm Transwell chambers (Corning), with 600 µL complete medium in the lower compartment. Following a 24-hour incubation period, non-invasive cells were gently eliminated. Transmigrated cells underwent fixation using 4% paraformaldehyde, labeled with 0.5% crystal violet solution, and quantified by light microscopy.

### Statistical analysis

The experiments were repeated independently a minimum of three replicates and presented as mean values ± SD. All statistical evaluations and computations were conducted using R software (4.4.0). Statistical significance was determined using unpaired t-tests and two-factor variance analyses (GraphPad Prism 8). Threshold for statistical significance was set at p<0.05.

## Result

### Identification of 11 important DEGs in ccRCC

**Figure 1:**
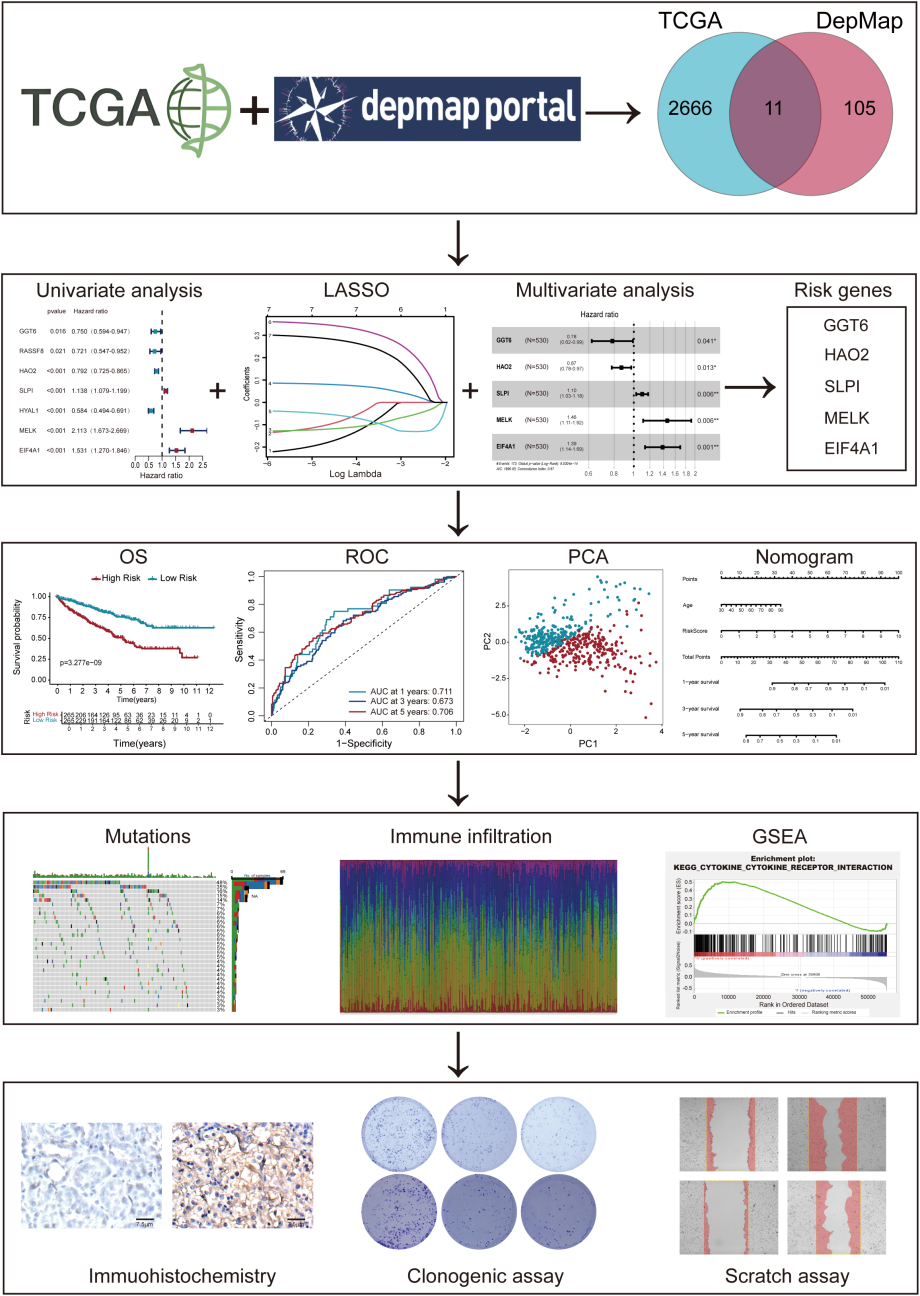
The flowchart and graphic abstract of this study.

**Figure 2:**
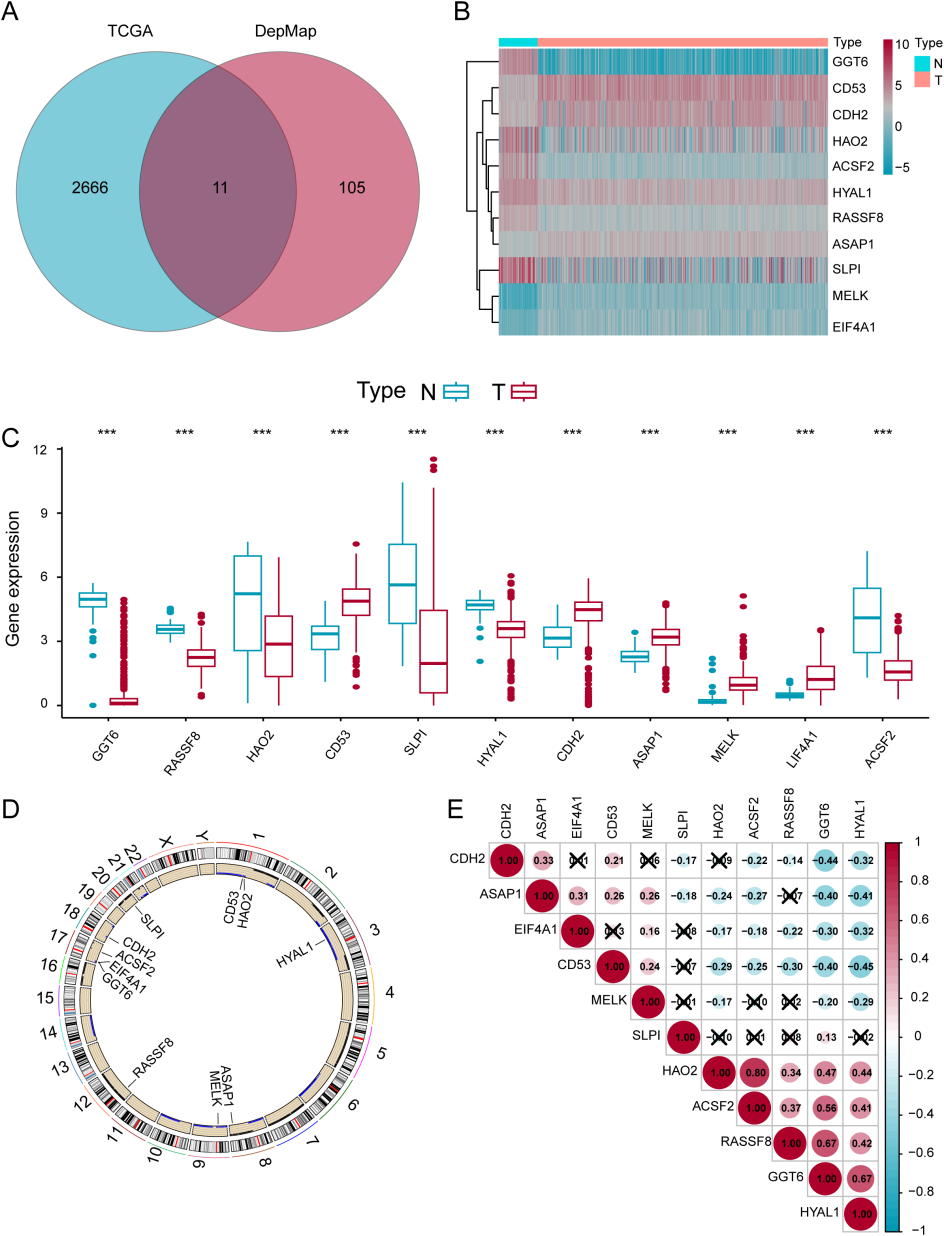
Identification of 11 Important DEGs in ccRCC. **(A)** Venn diagram of genes in the TCGA and DEPMap datasets. **(B)** Expression heatmap of the eleven genes in normal versus tumor samples. **(C)** Differential expression levels of the eleven genes in normal and tumor samples. **(D)** Locations of the DEGs on chromosomes. **(E)** Expression correlation analysis of the eleven DEGs. *p < 0.05; **p < 0.01; ***p < 0.001.

The complete analytical workflow is presented (**Figure 1**). Initially, essential genes that significantly impact cell viability in ccRCC cell lines were identified based on genome-wide CRISPR knockout screening data from the DepMap database. Subsequently, DEGs in ccRCC tumors compared to adjacent normal tissues were detected via TCGA transcriptomic data mining. By integrating these two datasets (**Figures 2A**), we identified 11 key genes exhibiting significant difference in ccRCC (**Figures 2B, C**). Further analysis revealed that these genes commonly exhibit CNVs, predominantly characterized by copy number losses (**Figure 2D**). Additionally, the correlations among these 11 DEGs are shown in **Figure 2E**. Most importantly, we successfully identified 11 crucial DEGs for further in-depth analysis.

### The construction and evaluation of the prognostic model

**Figure 3:**
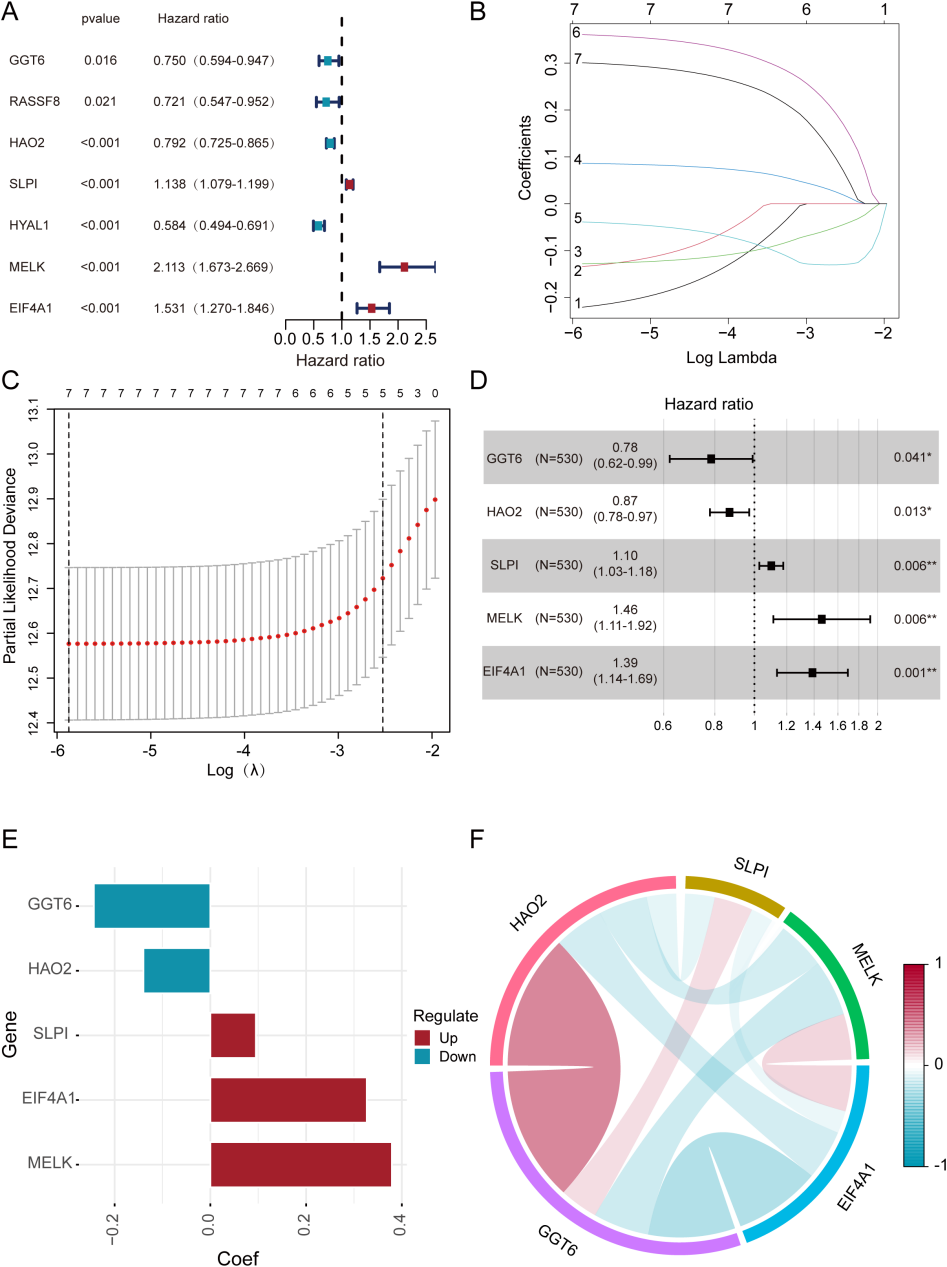
The construction and evaluation of the prognostic models. **(A)** Univariate Cox regression identifies 7 DEGs. **(B)** Coefficient trajectories of 7 DEGs in LASSO regression. **(C)** Optimal lambda selection in LASSO regression (10-fold CV). **(D, E)** Prognostic impact of 5 DEGs assessed by multivariate Cox regression. **(F)** Inter-gene correlations among the five DEGs.

Through univariate Cox regression analysis of the 11 candidate genes, we identified 7 genes that exhibited stronger associations with the prognosis of ccRCC. Subsequently, we employed the k-means clustering algorithm to perform grouping experiments on these 7 genes. The results demonstrated that the clustering performance was most stable when k=2 (**Supplementary Figures S1A–D**). UAMP revealed distinct gene expression patterns between cluster 1 and cluster 2 (**Supplementary Figure S1E**). Additionally, the Kaplan–Meier analysis demonstrated significantly better OS in cluster 2 compared to cluster 1 among ccRCC patients (**Supplementary Figure S1F**). The findings not only confirmed the classification of ccRCC patients into two subgroups but also revealed notable disparities in their OS. Pronounced differences in expression patterns between the two gene groups with high internal consistency. In the initial stage of our analysis, we performed univariate Cox regression on the 11 DEGs (**Figure 3A**). Subsequently, we applied LASSO regression to further refine the gene set (**Figures 3B, C**). Intriguingly, 7 genes were retained based on partial likelihood minimization and were subsequently applied in constructing the risk prediction model. Then we utilize multivariate Cox regression analysis, ultimately screening out 5 core genes: GGT6 (95% CI = 0.62-0.99, *p* = 0.041), HAO2 (95% CI = 0.78-0.97, *p* = 0.013), SLPI (95% CI = 1.03-1.18, *p* = 0.006), MELK (95% CI = 1.11-1.92, *p* = 0.006), and EIF4A1 (95% CI = 1.14-1.69, *p* = 0.001). These genes showed significant correlations with the OS (**Figures 3D, E**). The correlations between these DEGs are displayed (**Figure 3F**).

### Clinical evaluation based on a risk score-derived prognostic model

We built a risk score model from the transcriptional signatures of the five genes, dividing patients into high-risk and low-risk groups. Through heatmap analysis (**Supplementary Figure S2A**), we revealed potential associations between risk scores of ccRCC and clinical characteristics of patients. The heatmap results demonstrated a positive correlation between elevated risk scores and poor prognosis. To further quantify these relationships, we constructed scatter plots using the Wilcoxon signed-rank test (**Supplementary Figures S2B–G**). It indicated that ccRCC risk stratification exhibited a strong positive association with clinical stage, N stage, T stage, M stage, gender, and tumor grade (*p* < 0.05). However, no statistically significant correlation was observed between age and ccRCC risk scores (**Supplementary Figure S2H**). In summary, the ccRCC risk score serves as a robust indicator for evaluating tumor malignancy, with predictive efficacy independent of age.

### Prognostic stratification and risk assessment

**Figure 4:**
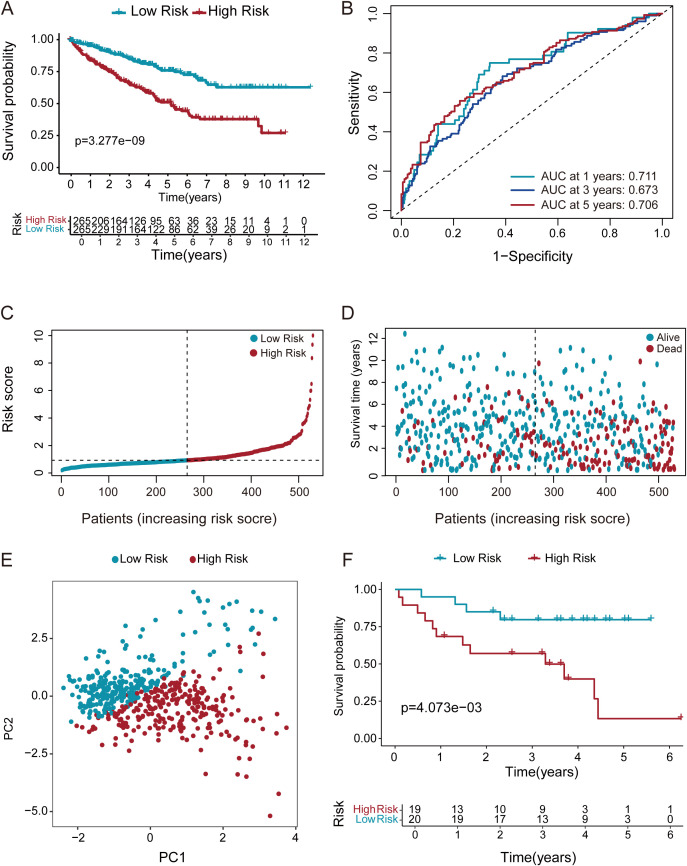
Multi method validation of risk score-derived prognostic models. **(A)** KM survival curves demonstrated markedly shorter overall survival in high-risk ccRCC patients relative to those in the low-risk group. **(B)** ROC analysis of the DEGs prognostic signature for predicting the 1/3/5-year survival. **(C, D)** Risk score stratification and survival duration distribution in ccRCC cohort. **(E)** PCA discriminates high- and low-risk groups using whole transcriptome data. **(F)** KM survival analysis of ccRCC patients stratified by risk score in the GEO validation cohort (GSE26909, n=39).

KM analysis confirmed a worse prognosis in high-risk versus low-risk patients (**Figure 4A**). Additionally, the prognostic value of our model was examined using ROC curve methodology (**Figure 4B**). The model demonstrated strong predictive accuracy with 1-year, 3-year, and 5-year AUCs of 0.711, 0.673, and 0.706, confirming its robust prognostic value. It’s displays the risk score distribution across high- and low-risk groups (**Figure 4C**). indicating a direct relationship between rising risk scores and mortality probability (**Figure 4D**). Furthermore, PCA was employed to classify ccRCC samples into distinct groups. PCA results distinctly stratified ccRCC samples into high-risk and low-risk groups, reaffirming the significant prognostic differentiation of ccRCC patients based on our risk model (**Figures 4E**). To further validate our prognostic model, we applied it to an independent GEO dataset (GSE26909, n=39). Consistent with TCGA results, the model significantly stratified patients into high- and low-risk groups (**Figure 4F**), confirming its generalizability.

### Formulation and evaluation of the nomogram

**Figure 5:**
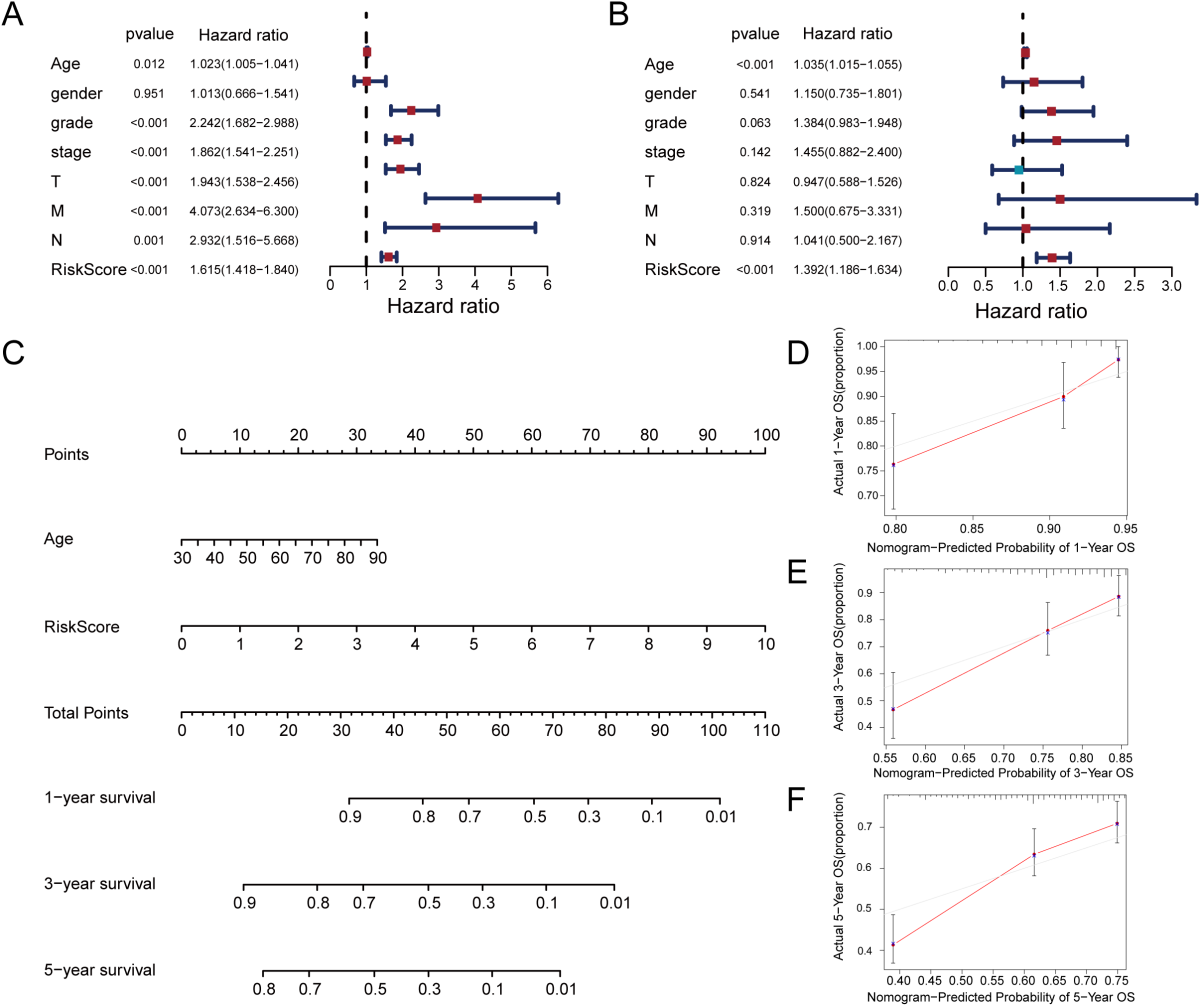
Construction of a nomogram for prediction prognosis. **(A)** Univariate Cox regression analysis identified grade, stage, T stage, M stage, and risk score as significant prognostic factors. **(B)** Multivariate Cox regression identifies risk score and age as independent prognostic predictors. **(C)** Prognostic nomogram incorporating risk score and age for ccRCC survival probability. **(D–F)** Calibration curves demonstrate the accuracy of 1-year, 3-year, and 5-year overall survival predictions.

Univariate and multivariate Cox proportional hazards models were utilized to evaluate the risk score’s independence as a prognostic indicator for ccRCC (**Figures 5A, B**). Notably, while age did not show a significant correlation with the risk score (**Supplementary Figure S2H**), multivariate Cox regression analysis confirmed its independent prognostic value for overall survival. Therefore, we included age in the nomogram and considered potential confounding factors, such as treatment tolerance and comorbidities, which may independently affect patient prognosis regardless of molecular risk stratification. Based on significant *p*-values from multivariate Cox regression, we constructed a nomogram as a quantitative method to predict OS in ccRCC patients(**Figure 5C**). The predictive factors included in the nomogram were the risk score and age. The results showed that the risk score was the key prognostic indicator. Additionally, calibration curves for 1-year, 3-year, and 5-year predictions were generated, demonstrating that the model exhibited satisfactory predictive accuracy (**Figures 5D–F**). The data indicate this signature may serve as a dependable assessment method for OS prediction in ccRCC.

### Investigating the relationship between TMB and risk scores

**Figure 6:**
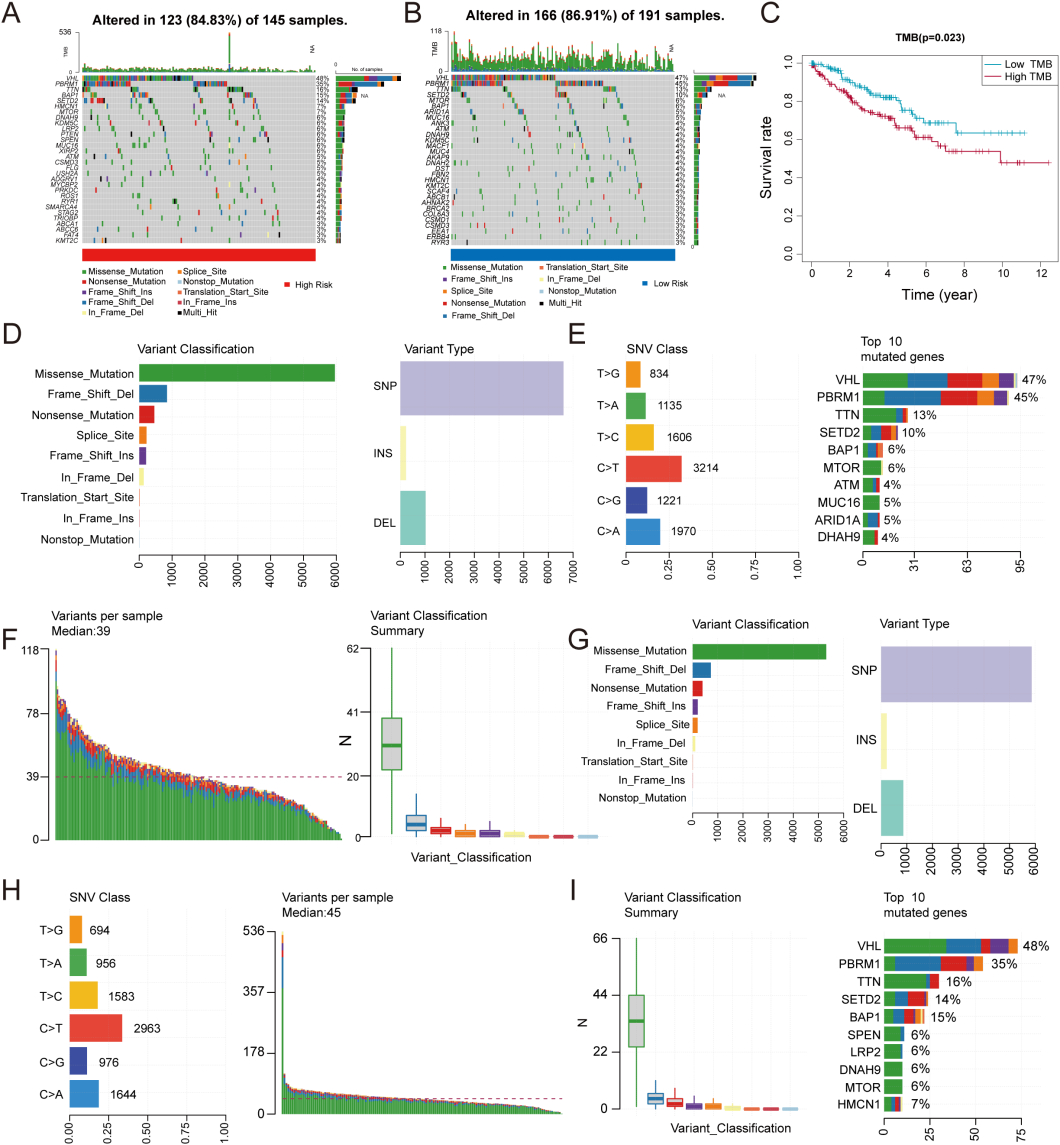
Correlation between TMB and risk score. **(A, B)** Comparative mutation landscapes in high-risk **(A)** and low-risk **(B)** groups. **(C)** Survival outcomes stratified by TMB levels. **(D–I)** Variant type distributions are shown for high-risk **(D–F)** and low-risk **(G–I)** patients.

Subsequently, we focused on the potential value of TMB in tumor immunotherapy and its molecular characteristics. We analyzed genomic alteration landscapes in high-risk and low-risk groups risk scores from the TCGA database (**Figures 6A, B**). Survival curves stratified by TMB levels indicated that patients with low TMB exhibited improved clinical prognosis compared to those with high TMB (**Figure 6C**). Subgroup analysis revealed significant differences in mutation distribution and genetic features between high TMB groups(**Figures 6D–F**) and low TMB groups(**Figures 6G–I**). Missense mutations predominated in both groups, while frameshift mutations demonstrated pronounced prevalence in the low TMB group, hinting at distinct functional impacts on tumor progression. Mutation distribution and gene characteristics also differed between TMB groups.

### Prognostic model using immune cells and drug sensitivity

**Figure 7:**
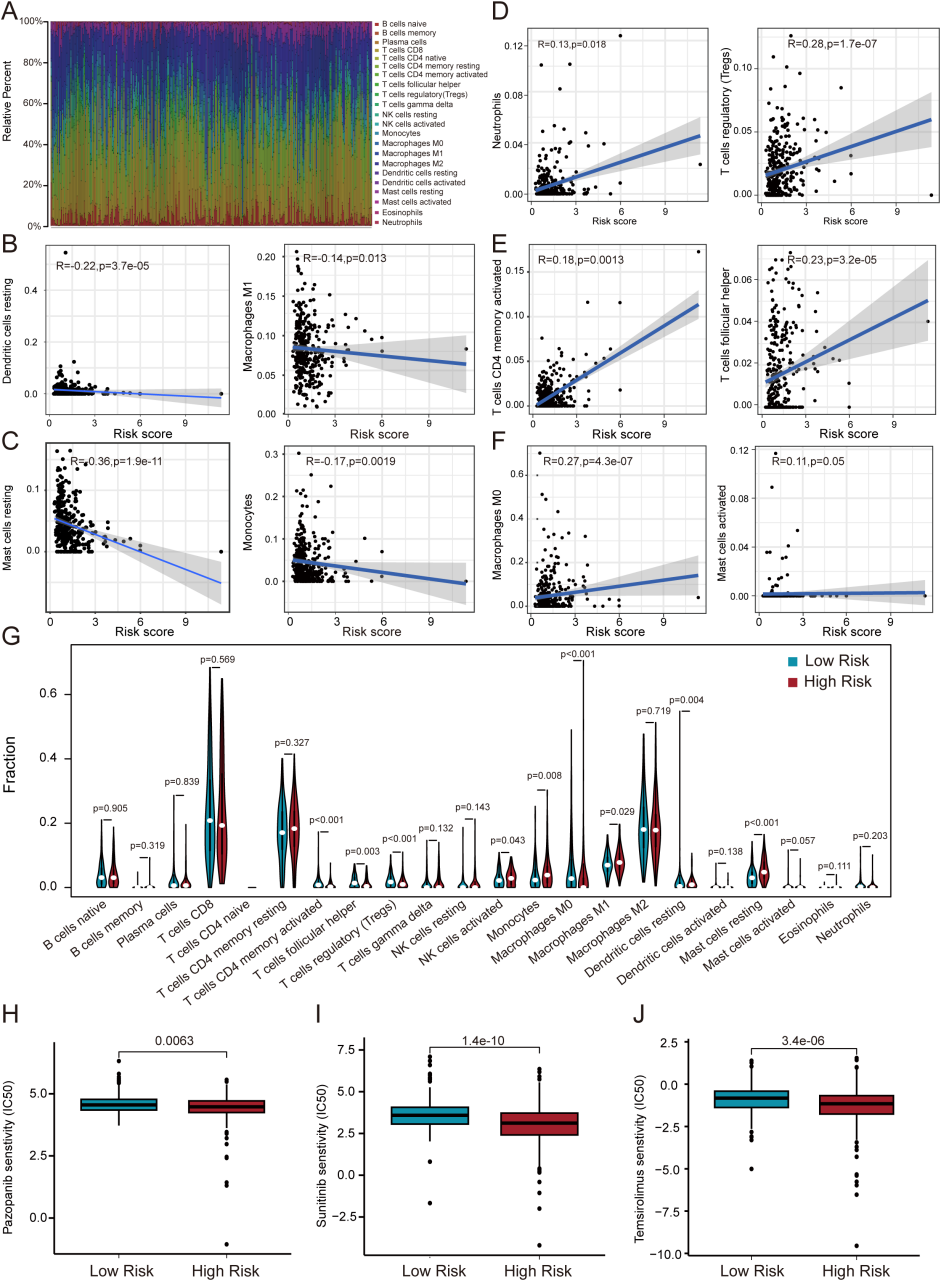
Correlation of immune microenvironment with risk score. **(A)** Immune cell infiltration landscape in ccRCC revealed by CIBERSORT. **(B–F)** Linear regression models demonstrate risk score-dependent immune cell infiltration patterns. **(G)** Differential immune cell distribution between risk groups. **(H–J)** Risk-stratified therapeutic sensitivity to pazopanib, sunitinib, and temsirolimus.

TME has been shown to have a critical impact on the progression and treatment of various cancers. By constructing an immune cell atlas of the TME, we systematically analyzed the infiltration patterns of 22 immune cell subsets in ccRCC (**Figure 7A**). Our findings revealed that immune cell populations including dendritic cells, M1 macrophages, mast cells, and monocytes exhibited significant anti-tumor activity, with their abundance positively correlated with improved patient prognosis (**Figures 7B, C**). In contrast, neutrophils, memory T cells, regulatory T cells, follicular helper T cells, M0 macrophages, activated mast cells demonstrated pro-tumor characteristics, and elevated infiltration levels correlated significantly with adverse clinical outcomes (**Figures 7D–F**). Further analysis using the ESTIMATE algorithm evaluated immune cell infiltration in the TME of ccRCC patients (**Figure 7G**). The results showed a marked reduction in anti-tumor immune cells and a concomitant increase in immunosuppressive cell infiltration in high-risk TME. Based on these derivations, we assessed the therapeutic efficacy of three targeted agents pazopanib, sunitinib, and temsirolimus in high-risk and low-risk group (**Figures 7H–J**). The research indicate that these agents show significantly higher drug sensitivity and improved treatment outcomes in low-risk patients. These findings indicate that our model is closely associated with tumor-infiltrating immune cells and drug sensitivity, providing valuable insights for the development of targeted immunotherapies in ccRCC.

### Enrichment analysis of the prognostic model

**Figure 8:**
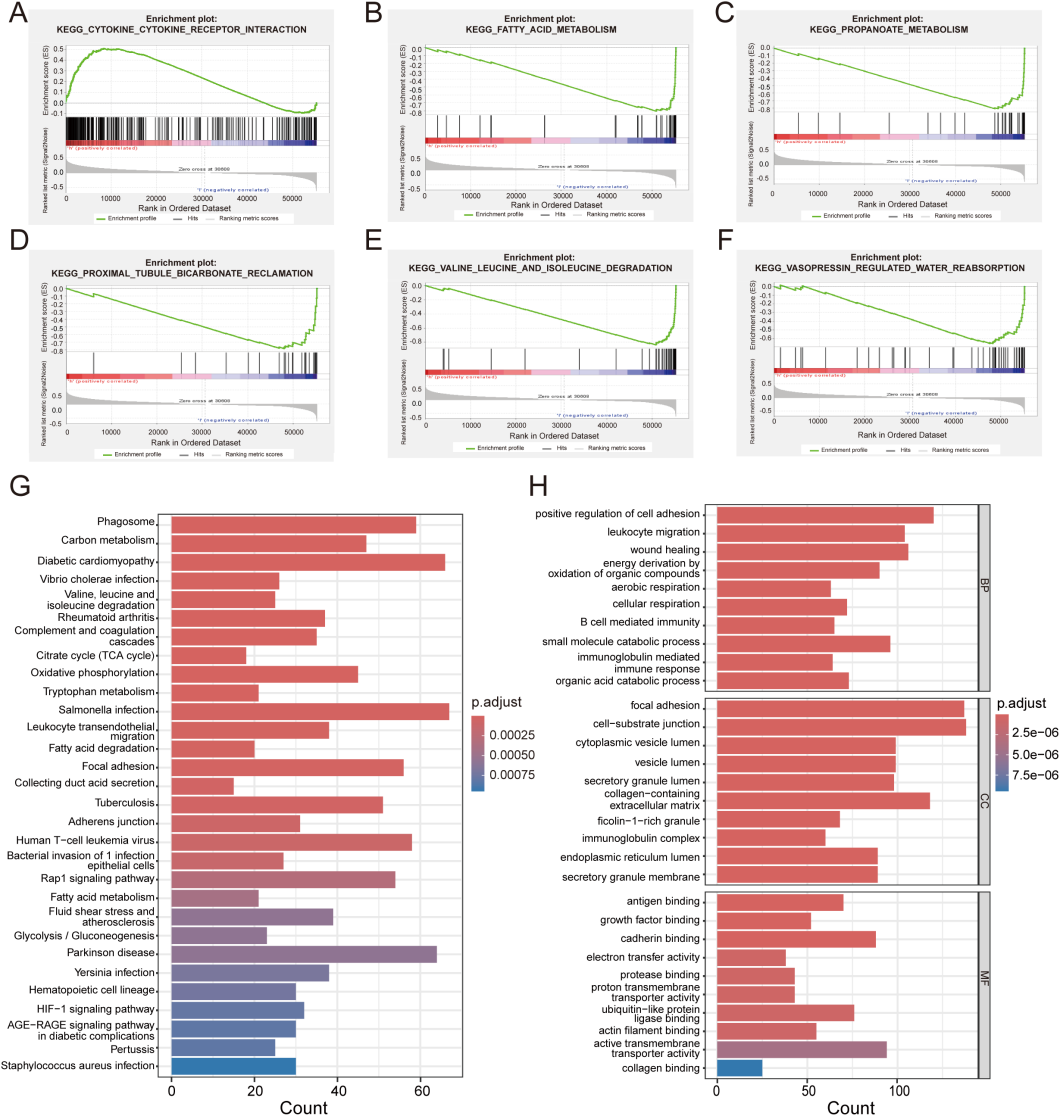
Functional enrichment and GSEA analysis. **(A)** Significantly enriched biological pathways in high-risk patients. **(B–F)** Distinct biological pathway enrichment profile in low-risk cohort. **(G)** GO analysis reveals key biological processes of DEGs. **(H)** KEGG pathway enrichment landscape of DEGs.

To further annotate the functional enrichments in the high-risk and low-risk groups, we performed GSEA to identify significantly enriched signaling pathways (**Figures 8A–F**). The high-risk group showed prominent enrichment in the “Cytokine-cytokine receptor interaction” pathway, while the low-risk group exhibited significant enrichment in metabolic pathways including fatty acid, propanoate, and branched-chain amino acid degradation. KEGG and GO analyses (**Figures 8G, H**) were performed to explore the molecular mechanisms of the five prognosis-related genes. KEGG pathway analysis indicated significant enrichments in pathways including Phagosome, Carbon metabolism, Diabetic cardiomyopathy. These findings suggest that the prognosis of RCC patients may be influenced by the aforementioned biological functions and signaling pathways. GO analysis highlighted enrichment in cell adhesion regulation, energy metabolism, and extracellular matrix components.

### High MELK expression is associated with poor prognosis in patients with ccRCC

**Figure 9:**
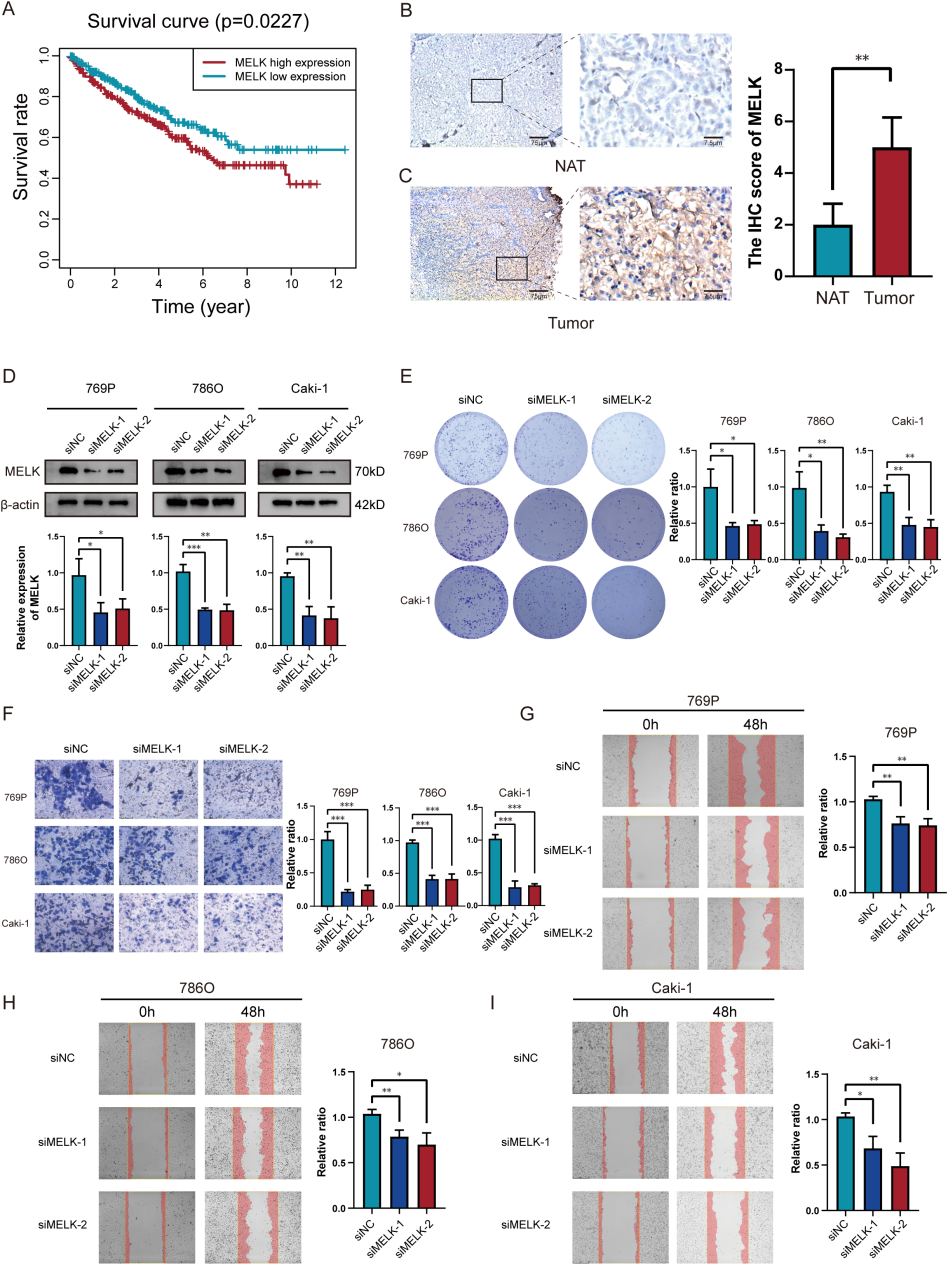
MELK is a poor prognostic marker in ccRCC. **(A)** Significant variations in overall survival between ccRCC patients with high and low MELK expression. **(B, C)** Immunohistochemical evidence of MELK overexpression in tumor tissues versus NAT. **(D)** Successful MELK knockdown confirmed by western blot across 769P, 786O and Caki-1 cell lines. **(E)** Silencing MELK suppressed proliferation abilities in 769P, 786O and Caki-1 cells. **(F–I)** Silencing MELK suppressed migration abilities as measured via transwell assay **(F)** and scratch assay **(G–I)** in 769P, 786O and Caki-1 cells. *p < 0.05; **p < 0.01; ***p < 0.001.

Based on existing studies, both MELK and EIF4A1 are highly expressed in tumor cells, and high EIF4A1 expression has been confirmed to correlate with poor patient prognosis (28). Elevated MELK (HR=1.46) and EIF4A1 (HR=1.39) expression predicted adverse outcomes, with MELK showing the highest risk association. Based on our analysis, high MELK expression levels correlated with adverse clinical outcomes (**Figure 9A**). IHC staining further demonstrated that MELK expression was higher in tumor tissues than in normal adjacent tissues (NAT) (**Figures 9B, C**), confirming that MELK levels are elevated in tumor tissues. Moreover, MELK levels increased significantly with tumor progression, showing higher expression in advanced-stage compared to early-stage ccRCC (**Supplementary Figures S3A–E**). Patients in the high-risk category demonstrated markedly elevated MELK expression compared to their low-risk counterparts. (**Supplementary Figure S3F**). MELK upregulation represents a potential prognostic marker in ccRCC. We selected three ccRCC cell lines (786-O, 769-P, and Caki-1) and transfected these cells with MELK-specific siRNA plasmids. Successful knockdown of MELK was confirmed by Western blotting (**Figure 9D**). MELK knockdown substantially inhibited colony formation and cell proliferation(**Figure 9E**). The results of migration assays demonstrated that relative counts of migrating cells were significantly reduced in MELK knockdown groups (**Figures 9F–I**). This indicates that MELK knockdown significantly suppresses the migratory abilities of 786-O, 769-P, and Caki-1 cells. Collectively, our clinical and experimental data establish MELK as a critical oncogenic driver in ccRCC, whose overexpression correlates with advanced tumor progression, poor prognosis, and enhanced malignant phenotypes, while its knockdown potently suppresses tumor aggressiveness, highlighting its potential as both a prognostic biomarker and therapeutic target.

## Discussion

As the predominant pathological category of renal carcinoma, ccRCC is notable for substantial heterogeneity and aggressive progression. Despite recent advancements in therapeutic strategies, the prognosis for ccRCC remains poor, particularly for advanced-stage patients (29, 30). Identifying key prognostic genes and constructing robust prognostic models are therefore critical for improving survival rates and guiding personalized treatment (31). Current ccRCC risk stratification methods primarily rely on clinical and pathological features, lacking consideration of tumor molecular mechanisms and the immune microenvironment. This limits their predictive accuracy and ability to provide personalized treatment recommendations. Our study integrates CRISPR-Cas9 gene-editing data from DepMap and transcriptome data from TCGA to construct a prognostic model, which has been further validated in an independent GEO cohort (GSE29609). This model not only enhances the accuracy of risk stratification but also offers more precise clinical guidance through drug sensitivity analysis. The consistent performance across multiple datasets (TCGA and GEO) demonstrates its robustness and generalizability. It helps optimize treatment plans, improve therapeutic outcomes, and reduce medical costs. The DepMap database, a comprehensive resource cataloging genetic dependencies in cancer cell lines, facilitated the identification of genes essential for ccRCC survival through CRISPR-Cas9 knockout screening. By leveraging DepMap’s Chronos scores we prioritized genes with significant functional relevance, ensuring that findings were grounded in both *in vitro* experimentation and clinical data (32). This dual-validation approach minimized false-positive results and enhanced the translational potential of the prognostic model. The development of genome-wide CRISPR-Cas9 loss-of-function screening represents a major breakthrough in biological research, offering a powerful tool to dissect gene function in tumorigenesis (33–37). Concurrently, TCGA project has unveiled the complex genomic landscape of ccRCC, including mutations, CNVs, dysregulated gene expression, and immune microenvironment alterations, laying the groundwork for novel diagnostic markers and therapeutic targets. This study integrates TCGA-derived ccRCC data with DepMap CRISPR-Cas9 screening to identify prognostic genes and construct a predictive model, thereby advancing precision medicine strategies for ccRCC.

From DepMap (CERES scores), we identified 116 ccRCC-essential proliferation genes, while TCGA-KIRC analysis uncovered 2,677 DEGs. Intersecting these datasets yielded 11 candidate genes. Subsequent univariate Cox and LASSO regression analyses narrowed the selection to five key genes—GGT6, HAO2, SLPI, MELK, and EIF4A1—whose expression patterns correlated strongly with tumor grade, clinical stage, and metastatic status. KM analysis revealed pronounced survival differences between gene-stratified high-risk and low-risk groups. ROC analysis confirmed the model’s superior predictive accuracy compared to conventional clinical parameters (AUC >0.75 for 1–5-year survival), while its age independence underscored its applicability across diverse patient populations. The prognostic model, validated by nomogram calibration and marked survival differences between risk groups, exhibited exceptional performance. Notably, MELK and EIF4A1 were highly expressed in tumor cells. MELK, a serine/threonine kinase implicated in cancer stem cell maintenance and chemoresistance in multiple malignancies, was associated with poor prognosis (38). Similarly, EIF4A1, a translation initiation factor, may drive tumor proliferation by enhancing oncoprotein synthesis, a mechanism observed in other cancers (39).

Further analysis revealed interactions between risk scores and TMB, highlighting their combined prognostic value. Patients with low TMB exhibited improved clinical outcomes, while distinct mutational profiles between high-TMB and low-TMB groups (e.g., VHL mutations in high TMB vs. DNAH9 in low TMB) emphasized the genomic heterogeneity of ccRCC and the need for tailored therapies. TME analysis demonstrated that immune cell infiltration patterns significantly influenced disease progression and treatment response. Anti-tumor immune cells, such as dendritic cells and M1 macrophages, were enriched in low-risk groups, whereas neutrophils and regulatory T cells (Tregs) exhibited pro-tumor activity (40). The immunosuppressive TME in high-risk patients, marked by reduced anti-tumor immunity and increased immunosuppressive cell infiltration, underscores the therapeutic potential of targeting the TME. Drug sensitivity assays validated the model’s clinical utility, revealing significant associations with pazopanib, sunitinib, and temsirolimus—agents targeting angiogenesis and mTOR pathways central to ccRCC treatment (41). Enrichment of “cytokine-cytokine receptor interaction” pathways in high-risk tumors further supports the potential of immunomodulatory therapies to counteract pro-tumor inflammation.

GSEA uncovered divergent signaling pathways between risk groups. High-risk patients exhibited enrichment in cytokine-related pathways linked to tumor progression and immune evasion, while low-risk patients showed metabolic pathway activation, suggesting metabolic reprogramming contributes to favorable outcomes. These findings deepen our understanding of ccRCC biology and highlight actionable therapeutic targets. For instance, HAO2, associated with fatty acid metabolism, underscores the role of metabolic dysregulation in driving tumor aggressiveness—a hallmark of ccRCC. HAO2 (glycine oxidase 2) is upregulated in ccRCC and involved in glycine oxidation, impacting cellular energy metabolism and oxidative stress response. Its overexpression may enhance tumor cell proliferation and survival by boosting energy metabolism and antioxidant capacity. Additionally, metabolic pathway alterations can influence immune cell infiltration in the tumor microenvironment, affecting tumor immune evasion (42).

This study establishes a multi-omics-driven prognostic framework for ccRCC, bridging genetic vulnerabilities with clinical outcomes. The identified genes and pathways not only enhance our mechanistic understanding of ccRCC but also offer translatable strategies for risk stratification and therapeutic innovation. MELK has been pinpointed as a core gene within the constructed prognostic model, playing a pivotal role in the genesis and progression of ccRCC. As a member of the AMPK-related kinase family, MELK is overexpressed in various malignancies including breast cancer, hepatocellular carcinoma, and glioma, where it drives oncogenesis by regulating cell cycle progression, cancer stemness, and therapy resistance (43, 44). Previous studies have demonstrated that MELK is not only crucial for the development of breast and liver cancers, but also contributes to radio- and chemoresistance in patients with hepatocellular carcinoma and glioma (45). Given its oncogenic properties, MELK is currently being investigated as a potential therapeutic target, although its specific impact on ccRCC requires further elucidation. Further validation studies in independent cohorts are warranted to confirm these observations, elucidating downstream signaling mechanisms, and exploring targeted therapies against MELK and EIF4A1 to realize their clinical potential. Among the five prognostic genes, MELK emerged as a central player in ccRCC progression. Our functional studies demonstrated that MELK knockdown potently inhibited tumor cell proliferation, migration and invasion in ccRCC cell lines. These results corroborate prior findings in other cancers, where MELK overexpression promotes tumorigenesis via cell cycle regulation and DNA damage repair. The elevated MELK expression in advanced-stage tumors and its correlation with poor prognosis highlight its potential as a therapeutic target. Notably, the efficacy of pazopanib, sunitinib, and temsirolimus in high-risk tumors suggests that targeting MELK-related pathways may synergize with existing therapies to improve outcomes.

Despite these advances, Certain methodological constraints merit careful consideration. First, the reliance on TCGA data may introduce selection bias, and external validation in independent cohorts is essential to confirm the model’s generalizability. Second, while *in vitro* experiments demonstrated MELK’s functional role, *in vivo* studies and mechanistic investigations are needed to elucidate its downstream signaling networks. Third, the clinical utility of the nomogram requires prospective validation to assess its impact on therapeutic decision-making.

Future studies should focus on translating these findings into clinical practice. For instance, exploring small-molecule inhibitors targeting MELK or EIF4A1 may open new avenues for precision therapy. Additionally, integrating immune cell infiltration profiles with genomic data could refine immunotherapy selection, particularly for patients with high-risk scores and immunosuppressive TME features.

In conclusion, our study has developed a novel prognostic framework for ccRCC by integrating CRISPR-Cas9 screening data from DepMap and transcriptomic profiles from TCGA. This approach bridges genomic vulnerabilities with clinical outcomes, offering a more comprehensive understanding of ccRCC biology compared to previous models that rely solely on transcriptomic data. The identified genes and pathways not only enhance our insights into the disease but also provide actionable targets for risk stratification and therapeutic development. Furthermore, the identification of MELK as a key driver gene and its association with the immunosuppressive tumor microenvironment highlight new avenues for targeted therapy in high-risk patients. Future validation and functional studies will be critical to realizing the translational potential of these findings and further improving the reliability and clinical applicability of our model.

## Data Availability

The original contributions presented in the study are included in the article/**Supplementary Material**. Further inquiries can be directed to the corresponding authors.

## Supplementary material

Supplementary Figure 1 Consensus clusters by 7 DEGs. **(A)** Cumulative distribution function (CDF) plot demonstrating consensus clustering stability. **(B)** Delta area plot showing relative changes in CDF curve area for each k value. **(C)** Consensus clustering matrix identifies two distinct molecular subtypes (k=2). **(D)** Cluster stability assessment. **(E)** Transcriptome-wide UAMP reveals inter-cluster divergence. **(F)** Survival disparity between clusters by KM analysis.

Supplementary Figure 2 Clinical Evaluation Based on a Risk Score-Derived Prognostic Models. The heatmap **(A)** and scatter plots demonstrate association of the stage **(B)**, N stage **(C)**, T stage **(D)**, M stage **(E)**, gender **(F)**, grade **(G)**, and age **(H)** with the risk score.

Supplementary Figure 3 Expression levels and functions of the MELK gene. Box plots of MELK gene expression in different clinical stages. Scatter plots demonstrate that T stage **(A)**, N stage **(B)**, M stage **(C)**, grade **(D)**, and stage **(E)**.Violin plots show the differential expression of the MELK gene between the high - risk and low - risk groups **(F)**.

## References

[1] Znaor A Lortet-Tieulent J Laversanne M Jemal A Bray F. International variations and trends in renal cell carcinoma incidence and mortality. Eur Urol. (2015) 67:519–30. doi: 10.1016/j.eururo.2014.10.002, PMID: 25449206

[2] Li G Xie ZK Zhu DS Guo T Cai QL Wang Y. KIF20B promotes the progression of clear cell renal cell carcinoma by stimulating cell proliferation. J Cell Physiol. (2019) 234:16517–25. doi: 10.1002/jcp.28322, PMID: 30805928

[3] Hsieh JJ Purdue MP Signoretti S Swanton C Albiges L Schmidinger M. Renal cell carcinoma. Nat Rev Dis Primers. (2017) 3:17009. doi: 10.1038/nrdp.2017.9, PMID: 28276433 PMC5936048

[4] Zhai W Zhu R Ma J Gong D Zhang H Zhang J. A positive feed-forward loop between LncRNA-URRCC and EGFL7/P-AKT/FOXO3 signaling promotes proliferation and metastasis of clear cell renal cell carcinoma. Mol Cancer. (2019) 18:81. doi: 10.1186/s12943-019-0998-y, PMID: 30953521 PMC6449923

[5] Doudna JA Charpentier E. Genome editing. The new frontier of genome engineering with CRISPR-Cas9. Science. (2014) 346:1258096. doi: 10.1126/science.1258096, PMID: 25430774

[6] Kurata M Yamamoto K Moriarity BS Kitagawa M Largaespada DA. CRISPR/Cas9 library screening for drug target discovery. J Hum Genet. (2018) 63:179–86. doi: 10.1038/s10038-017-0376-9, PMID: 29158600

[7] Sun X Wang Z Chen X Shen K. CRISPR-cas9 screening identified lethal genes enriched in cell cycle pathway and of prognosis significance in breast cancer. Front Cell Dev Biol. (2021) 9:646774. doi: 10.3389/fcell.2021.646774, PMID: 33816496 PMC8017240

[8] Ansori AN Antonius Y Susilo RJ Hayaza S Kharisma VD Parikesit AA. Application of CRISPR-Cas9 genome editing technology in various fields: A review. Narra J. (2023) 3:e184. doi: 10.52225/narra.v3i2.184, PMID: 38450259 PMC10916045

[9] Stefanoudakis D Kathuria-Prakash N Sun AW Abel M Drolen CE Ashbaugh C. The potential revolution of cancer treatment with CRISPR technology. Cancers (Basel). (2023) 15. doi: 10.3390/cancers15061813, PMID: 36980699 PMC10046289

[10] Meyers RM Bryan JG Mcfarland JM Weir BA Sizemore AE Xu H. Computational correction of copy number effect improves specificity of CRISPR-Cas9 essentiality screens in cancer cells. Nat Genet. (2017) 49:1779–84. doi: 10.1038/ng.3984, PMID: 29083409 PMC5709193

[11] Li T Zhang C Zhao G Zhang X Hao M Hassan S. Data analysis of PD-1 antibody in the treatment of melanoma patients. Data Brief. (2020) 30:105523. doi: 10.1016/j.dib.2020.105523, PMID: 32322636 PMC7168734

[12] Whitehead MJ Mccanney GA Willison HJ Barnett SC. MyelinJ: an ImageJ macro for high throughput analysis of myelinating cultures. Bioinformatics. (2019) 35:4528–30. doi: 10.1093/bioinformatics/btz403, PMID: 31095292 PMC6821319

[13] Wang H Lengerich BJ Aragam B Xing EP. Precision Lasso: accounting for correlations and linear dependencies in high-dimensional genomic data. Bioinformatics. (2019) 35:1181–7. doi: 10.1093/bioinformatics/bty750, PMID: 30184048 PMC6449749

[14] Tibshirani R. The lasso method for variable selection in the Cox model. Stat Med. (1997) 16:385–95. doi: 10.1002/(SICI)1097-0258(19970228)16:4<385::AID-SIM380>3.0.CO;2-3 9044528

[15] Cao R Wu Q Li Q Yao M Zhou H. A 3-mRNA-based prognostic signature of survival in oral squamous cell carcinoma. PeerJ. (2019) 7:e7360. doi: 10.7717/peerj.7360, PMID: 31396442 PMC6679650

[16] Yang S Wu Y Deng Y Zhou L Yang P Zheng Y. Identification of a prognostic immune signature for cervical cancer to predict survival and response to immune checkpoint inhibitors. Oncoimmunology. (2019) 8:e1659094. doi: 10.1080/2162402X.2019.1659094, PMID: 31741756 PMC6844304

[17] Lorent M Giral M Foucher Y. Net time-dependent ROC curves: a solution for evaluating the accuracy of a marker to predict disease-related mortality. Stat Med. (2014) 33:2379–89. doi: 10.1002/sim.6079, PMID: 24399671

[18] Harrell FE Jr. Lee KL Mark DB. Multivariable prognostic models: issues in developing models, evaluating assumptions and adequacy, and measuring and reducing errors. Stat Med. (1996) 15:361–87. doi: 10.1002/(SICI)1097-0258(19960229)15:4<361::AID-SIM168>3.0.CO;2-4, PMID: 8668867

[19] Kim S Park JM Rhyu S Nam J Lee K. Quantitative analysis of piano performance proficiency focusing on difference between hands. PloS One. (2021) 16:e0250299. doi: 10.1371/journal.pone.0250299, PMID: 34010289 PMC8133499

[20] Wilkerson MD Hayes DN. ConsensusClusterPlus: a class discovery tool with confidence assessments and item tracking. Bioinformatics. (2010) 26:1572–3. doi: 10.1093/bioinformatics/btq170, PMID: 20427518 PMC2881355

[21] Pond GR Agarwal N Bellmunt J Choueiri TK Qu A Fougeray R. A nomogram including baseline prognostic factors to estimate the activity of second-line therapy for advanced urothelial carcinoma. BJU Int. (2014) 113:E137–43. doi: 10.1111/bju.12564, PMID: 24219029

[22] Sun W Li G Song Y Zhu Z Yang Z Chen Y. A web based dynamic MANA Nomogram for predicting the Malignant cerebral edema in patients with large hemispheric infarction. BMC Neurol. (2020) 20:360. doi: 10.1186/s12883-020-01935-6, PMID: 32993551 PMC7523347

[23] Bakin EA Stanevich OV Danilenko DM Lioznov DA Kulikov AN. Fast prototyping of a local fuzzy search system for decision support and retraining of hospital staff during pandemic. Health Inf Sci Syst. (2021) 9:21. doi: 10.1007/s13755-021-00150-y, PMID: 33986947 PMC8112214

[24] Mayakonda A Lin DC Assenov Y Plass C Koeffler HP. Maftools: efficient and comprehensive analysis of somatic variants in cancer. Genome Res. (2018) 28:1747–56. doi: 10.1101/gr.239244.118, PMID: 30341162 PMC6211645

[25] Yu G Wang LG Han Y He QY. clusterProfiler: an R package for comparing biological themes among gene clusters. Omics. (2012) 16:284–7. doi: 10.1089/omi.2011.0118, PMID: 22455463 PMC3339379

[26] Sun X Wu K Cook D. PKgraph: an R package for graphically diagnosing population pharmacokinetic models. Comput Methods Programs BioMed. (2011) 104:461–71. doi: 10.1016/j.cmpb.2011.03.016, PMID: 21555161

[27] Geeleher P Cox N Huang RS. pRRophetic: an R package for prediction of clinical chemotherapeutic response from tumor gene expression levels. PloS One. (2014) 9:e107468. doi: 10.1371/journal.pone.0107468, PMID: 25229481 PMC4167990

[28] Zhang LL Chang W He SB Zhang B Ma G Shang PF. High expression of eIF4A1 predicts unfavorable prognosis in clear cell renal cell carcinoma. Mol Cell Probes. (2022) 65:101845. doi: 10.1016/j.mcp.2022.101845, PMID: 35820642

[29] Tan SK Hougen HY Merchan JR Gonzalgo ML Welford SM. Fatty acid metabolism reprogramming in ccRCC: mechanisms and potential targets. Nat Rev Urol. (2023) 20:48–60. doi: 10.1038/s41585-022-00654-6, PMID: 36192502 PMC10826284

[30] Linehan WM Ricketts CJ. The Cancer Genome Atlas of renal cell carcinoma: findings and clinical implications. Nat Rev Urol. (2019) 16:539–52. doi: 10.1038/s41585-019-0211-5, PMID: 31278395

[31] Sato Y Yoshizato T Shiraishi Y Maekawa S Okuno Y Kamura T. Integrated molecular analysis of clear-cell renal cell carcinoma. Nat Genet. (2013) 45:860–7. doi: 10.1038/ng.2699, PMID: 23797736

[32] Tsherniak A Vazquez F Montgomery PG Weir BA Kryukov G Cowley GS. Defining a cancer dependency map. Cell. (2017) 170:564–576.e16. doi: 10.1016/j.cell.2017.06.010, PMID: 28753430 PMC5667678

[33] Morgens DW Deans RM Li A Bassik MC. Systematic comparison of CRISPR/Cas9 and RNAi screens for essential genes. Nat Biotechnol. (2016) 34:634–6. doi: 10.1038/nbt.3567, PMID: 27159373 PMC4900911

[34] Onishi I Yamamoto K Kinowaki Y Kitagawa M Kurata M. To discover the efficient and novel drug targets in human cancers using CRISPR/Cas screening and databases. Int J Mol Sci. (2021) 22. doi: 10.3390/ijms222212322, PMID: 34830205 PMC8622676

[35] Gonçalves E Behan FM Louzada S Arnol D Stronach EA Yang F. Structural rearrangements generate cell-specific, gene-independent CRISPR-Cas9 loss of fitness effects. Genome Biol. (2019) 20:27. doi: 10.1186/s13059-019-1637-z, PMID: 30722791 PMC6362594

[36] Gonçalves E Thomas M Behan FM Picco G Pacini C Allen F. Minimal genome-wide human CRISPR-Cas9 library. Genome Biol. (2021) 22:40. doi: 10.1186/s13059-021-02268-4, PMID: 33478580 PMC7818936

[37] Sinha S Barbosa K Cheng K Leiserson MDM Jain P Deshpande A. A systematic genome-wide mapping of oncogenic mutation selection during CRISPR-Cas9 genome editing. Nat Commun. (2021) 12:6512. doi: 10.1038/s41467-021-26788-6, PMID: 34764240 PMC8586238

[38] Ren L Guo JS Li YH Dong G Li XY. Structural classification of MELK inhibitors and prospects for the treatment of tumor resistance: A review. BioMed Pharmacother. (2022) 156:113965. doi: 10.1016/j.biopha.2022.113965, PMID: 36411642

[39] Xia T Dai XY Sang MY Zhang X Xu F Wu J. IGF2BP2 drives cell cycle progression in triple-negative breast cancer by recruiting EIF4A1 to promote the m6A-modified CDK6 translation initiation process. Adv Sci (Weinh). (2024) 11:e2305142. doi: 10.1002/advs.202305142, PMID: 37983610 PMC10767445

[40] Fridman WH Zitvogel L Sautès-Fridman C Kroemer G. The immune contexture in cancer prognosis and treatment. Nat Rev Clin Oncol. (2017) 14:717–34. doi: 10.1038/nrclinonc.2017.101, PMID: 28741618

[41] Motzer RJ Escudier B Mcdermott DF Arén Frontera O Melichar B Powles T. Survival outcomes and independent response assessment with nivolumab plus ipilimumab versus sunitinib in patients with advanced renal cell carcinoma: 42-month follow-up of a randomized phase 3 clinical trial. J Immunother Cancer. (2020) 8. doi: 10.1136/jitc-2020-000891, PMID: 32661118 PMC7359377

[42] Liu M Pan Q Xiao R Yu Y Lu W Wang L. A cluster of metabolism-related genes predict prognosis and progression of clear cell renal cell carcinoma. Sci Rep. (2020) 10:12949. doi: 10.1038/s41598-020-67760-6, PMID: 32737333 PMC7395775

[43] Wang Y Lee YM Baitsch L Huang A Xiang Y Tong H. MELK is an oncogenic kinase essential for mitotic progression in basal-like breast cancer cells. Elife. (2014) 3:e01763. doi: 10.7554/eLife.01763.033, PMID: 24844244 PMC4059381

[44] Li Z Zhou H Zhai X Gao L Yang M An B. MELK promotes HCC carcinogenesis through modulating cuproptosis-related gene DLAT-mediated mitochondrial function. Cell Death Dis. (2023) 14:733. doi: 10.1038/s41419-023-06264-3, PMID: 37949877 PMC10638394

[45] Meel MH De Gooijer MC Guillén Navarro M Waranecki P Breur M Buil LCM. MELK inhibition in diffuse intrinsic pontine glioma. Clin Cancer Res. (2018) 24:5645–57. doi: 10.1158/1078-0432.CCR-18-0924, PMID: 30061363

