# Supplementary figures and images for: CRISPR/Cas9-based discovery of ccRCC therapeutic opportunities through molecular mechanism and immune microenvironment analysis
Source: Front Immunol. 2025 Jul 10;16:1619361. doi: 10.3389/fimmu.2025.1619361 (PMC12287010; doi:10.3389/fimmu.2025.1619361)

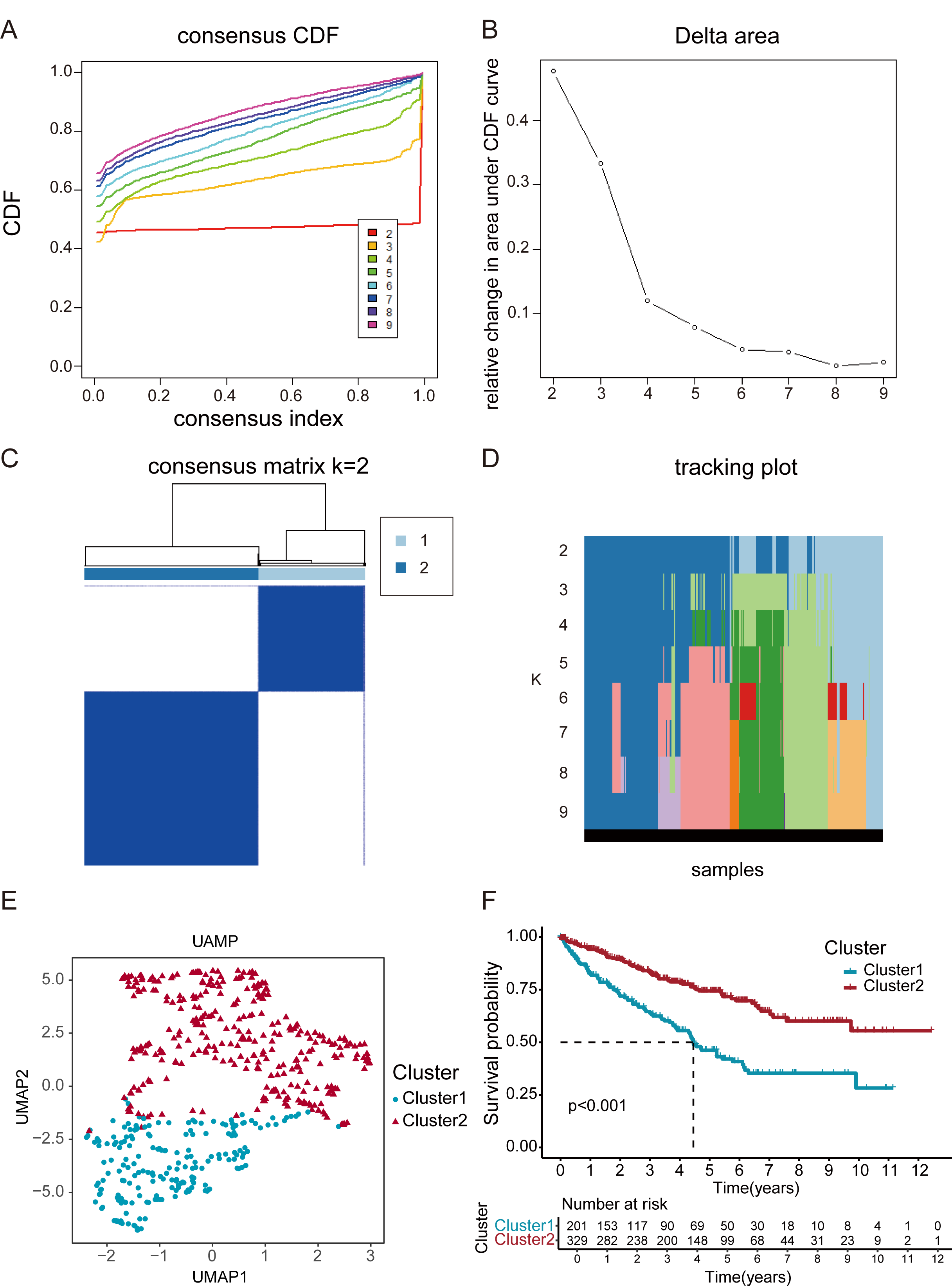

Supplement: Supplementary Figure 1 — Consensus clusters by 7 DEGs. (A) Cumulative distribution function (CDF) plot demonstrating consensus clustering stability. (B) Delta area plot showing relative changes in CDF curve area for each k value. (C) Consensus clustering matrix identifies two distinct molecular subtypes (k=2). (D) Cluster stability assessment. (E) Transcriptome-wide UAMP reveals inter-cluster divergence. (F) Survival disparity between clusters by KM analysis. [file Image1.tif]

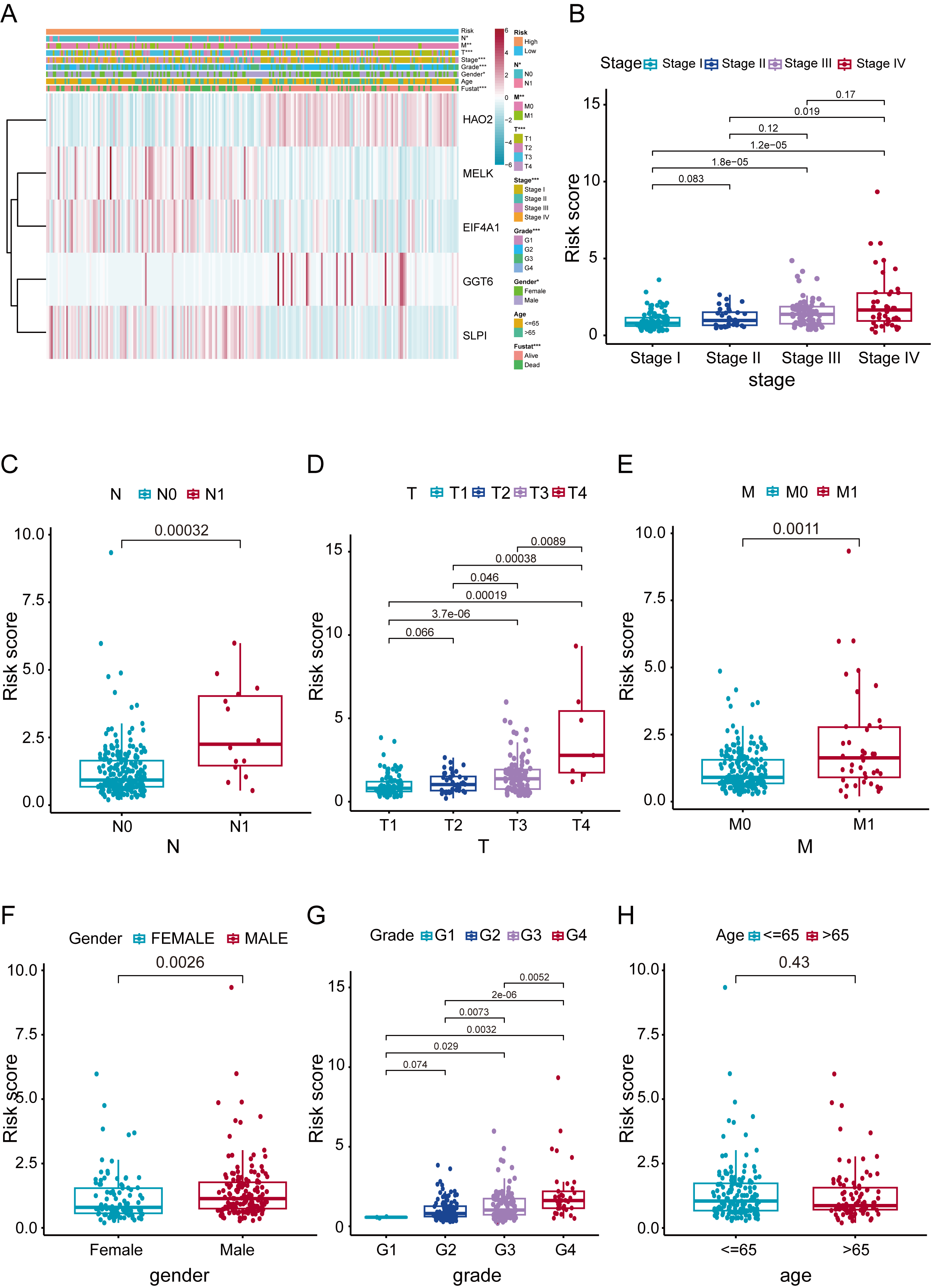

Supplement: Supplementary Figure 2 — Clinical Evaluation Based on a Risk Score-Derived Prognostic Models. The heatmap (A) and scatter plots demonstrate association of the stage (B), N stage (C), T stage (D), M stage (E), gender (F), grade (G), and age (H) with the risk score. [file Image2.tif]

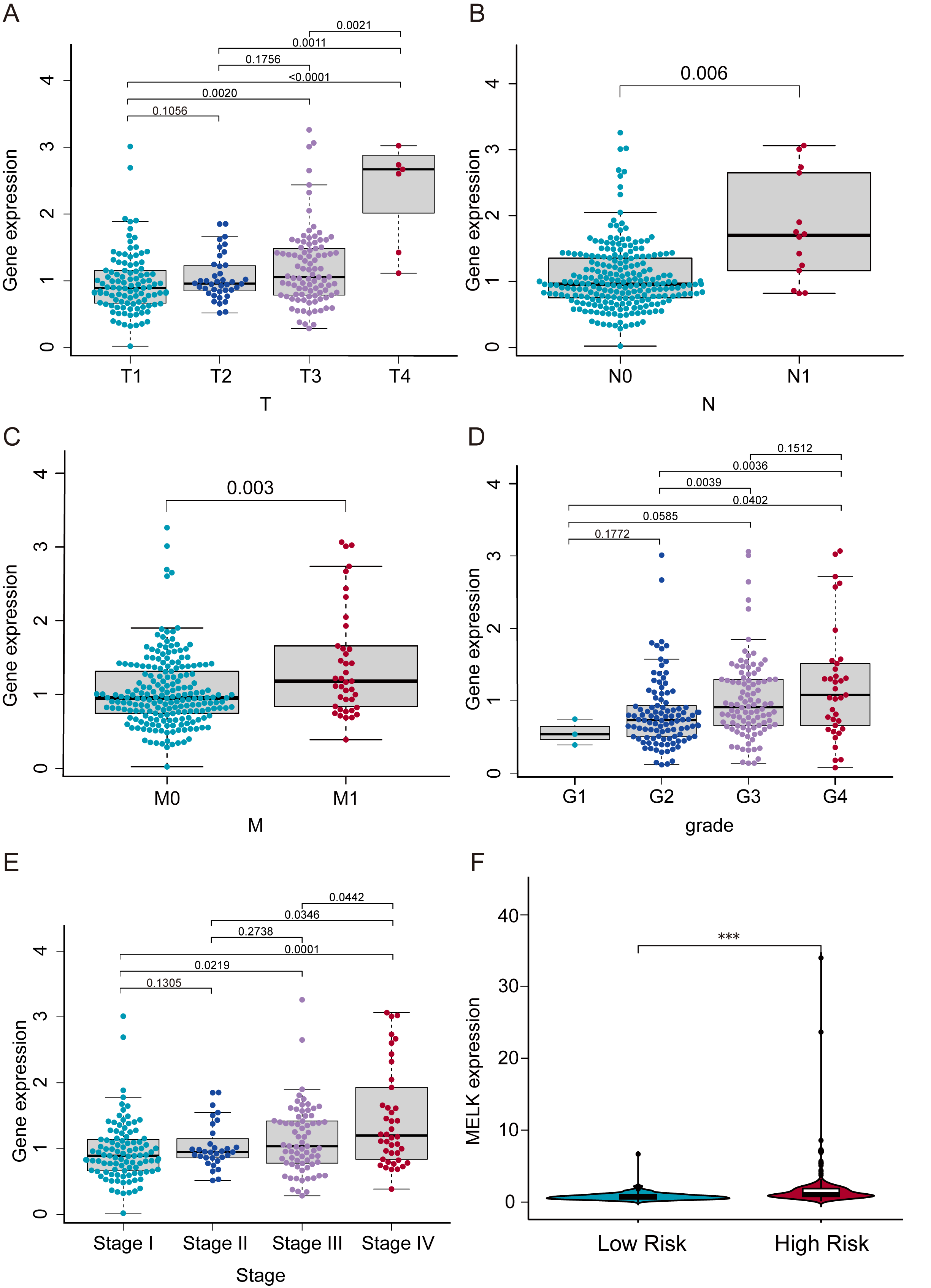

Supplement: Supplementary Figure 3 — Expression levels and functions of the MELK gene. Box plots of MELK gene expression in different clinical stages. Scatter plots demonstrate that T stage (A), N stage (B), M stage (C), grade (D), and stage (E).Violin plots show the differential expression of the MELK gene between the high - risk and low - risk groups (F). [file Image3.tif]
